# Moments of Pleasure: A Preliminary Classification of Gustatory *mmms* and the Enactment of Enjoyment During Infant Mealtimes

**DOI:** 10.3389/fpsyg.2019.01404

**Published:** 2019-07-02

**Authors:** Sally Wiggins

**Affiliations:** Department of Behavioral Sciences and Learning, Linköping University, Linköping, Sweden

**Keywords:** infants, gustatory *mmms*, enjoyment, eating, complementary feeding, discursive psychology

## Abstract

The enjoyment of food and the sharing of mealtimes is a normative cultural and social practice. Empirical research on eating enjoyment has, however, been a rather neglected area across the social sciences, often marginalized in favor of health or focusing on individual preferences rather than shared enjoyment. Even with regards to children, their enjoyment of food is typically rated retrospectively via parental reports of mealtime behavior. What is missing is an understanding of how enjoyment becomes a normative, cultural practice during mealtimes. This paper examines this issue in the context of parents feeding their 5–8-month-old infants in the family home, since it is within this context that we can see the early emergence of such practices in often highly routinized situations. The enactment of eating as enjoyable, and of the food as appreciated or “liked” in some way, is a culturally normative practice that becomes recognizable through particular non-lexical (“mmm,” “ooh”) or lexical (“this is nice, isn't it?”) utterances. The data comprise 66 infant mealtimes video-recorded over almost 19 h, from five families living in Scotland. The analysis uses discursive psychology and focuses on the sequential position of different types of parental gustatory *mmms* as produced during the infant meals. A classification of four types of *mmm* were identified in the corpus—announcement, receipting, modeling, and encouragement *mmms*—each associated with features of sequential and multimodal organization within the mealtime. In the majority of instances, *mmm*s were uttered alone with no other assessment terms, and parents typically produced these as an orientation to the enjoyment of their infants', rather than their own, eating practices. The receipting *mmms*, for instance, occurred at the precise moment when the infant's mouth closed around the food. It is argued that eating enjoyment can be considered as much an interactional practice as an individual sensation, and that non-lexical vocalizations around food are an essential part of sensory practices. The paper thus aims to bridge the gap between cultural and psychological studies of eating enjoyment and contribute to developmental studies of infant feeding in everyday interaction.

## Introduction

To enjoy one's food, and to share food with other people, can be one of the daily pleasures in life. We might not always enjoy what we eat, but there is a widespread assumption that enjoyment *should* play an important role in our consumption of food. Cultural greetings used when commencing eating orient toward enjoyment (e.g., “bon appétit” “smaklig”) and restaurant staff may ask customers if they enjoyed their meal. Despite this normative orientation to enjoyment, there is surprisingly little research that examines enjoyment or pleasure as these become relevant during eating practices. One could argue that health issues around food have been prioritized over enjoyment, and that the two categories (health, pleasure) have been treated as antagonistic to one another. The pleasurable aspects of eating have taken second place in empirical research. Where they have been studied, the focus on individual aspects of enjoyment and the related concepts of “liking” and “food preferences” have taken precedence. While sociological and anthropological work considers the social and cultural aspects of enjoyment, these too have received limited attention to date and have yet to examine *how* enjoyment becomes a social object.

To help redress this balance, the current paper examines one of the processes through which enjoyment becomes an interactional practice and a social phenomenon. While not denying individual dimensions of enjoyment, the aim instead is to examine those moments in which enjoyment is enacted, to begin to understand how food and eating *becomes* enjoyable. In particular, the focus is on those moments during infants' first experiences of solid foods (during “weaning” or “complementary feeding,” when they are around 5–8 months old), since it is here that they learn what it is to eat, not just to feed. As such, the paper aims to provide a bridge between cultural and individual perspectives on enjoyment. By focusing on the interaction between parent and child, enjoyment can be examined as a relevant social action that becomes available at key moments in eating practices. Moreover, this orientation is brought about primarily through an embodied, non-lexical vocalization—the gustatory *mmm*—and so provides a further contribution to work on the interactional organization of sensory practices.

### On Eating Enjoyment Across the Social Sciences

The theoretical promiscuity of “enjoyment” and its ability to traverse many disciplines—from philosophy to physiology—means that it is a rather fluid concept even though there are core features that are fairly consistent. With regards to eating, one can define enjoyment as an experience, sensation, or perception of food or eating practices that is positively evaluated in some way. There is often an assumed physiological element, and so enjoyment of eating is typically conceptualized as an individualized experience with its locus in the physical body. Its various synonyms—pleasure, hedonism, liking—have been more meticulously examined, and at times these are used interchangeably with enjoyment in the literature. The concept of hedonism, for example, while being centuries-old, only gained attention in psychological research on eating behavior after the apparent demise of behaviorist notions of reinforcement (Bolles, 2014). While clearly related to enjoyment, it is nevertheless important to distinguish these concepts, since—like “food preference”—research into taste hedonics has been more firmly situated within individual taste experiences and consumption behavior (e.g., Cox et al., 2016).

The fluidity of enjoyment is not in itself problematic, since it provides for a variety of research perspectives: some treat enjoyment as intellectual or social pleasure, for instance, rather than physiologically, or sensory-based. Nor is it always necessary to isolate a particular term (“enjoyment”) as distinctive from another (“pleasure”). What is argued, however, is that there is a tendency across the cultural and behavioral sciences to prioritize an individual locus of enjoyment at the expense of an interactional or social perspective (see also Wiggins, 2002). When “enjoyment” is used synonymously with “liking,” for instance, then there is a risk that only individual ratings or experiences of enjoyment will be studied. Problems arise when methodological practices do not match the theoretical assumptions. Even those who highlight the shared nature of enjoyment have yet to examine how this enjoyment becomes realized in specific social contexts.

The tendency toward an individualistic focus is compounded by the limited research that exists across psychology and the social sciences on the enjoyment of eating. The topic barely gets a mention in psychology of food and eating textbooks, unless as part of the pleasure vs. control dichotomy seen in models of health behavior and disordered eating (Ogden, 2010; see also next section). It has become marginalized as an emotional or affective response to eating. Psychologists have typically shied away from enjoyment as a topic that is too “subjective” for scientific studies of eating (though see research on recognition of a “genuine” enjoyment smile; Giudice and Colle, 2007). Even the broader area of taste, as one of the core senses through which we might experience enjoyment, has had a remarkably short analytical history. It was originally considered too closely related to carnal desires or relatively inaccessible in terms of shared experiences (McQuaid, 2015). We can make comparisons between what others see, hear or touch, for instance, but our tastes are seemingly more private and unique (cf. Spence, 2017). In short, psychologists have typically avoided the area of eating enjoyment, favoring instead the cognitivistic and physiological concepts of food preference or sensory hedonics and blurring the distinction between “liking” and “preference” (Mela, 2006). It is not pleasure that is typically being studied, but rather individual preferences for one type of food over another (Eccleston, 2016).

In sociology and anthropology, enjoyment of eating has also been largely overlooked (Warde and Martens, 2000; Warde, 2016), though here there is a broader consideration of social aspects, such as how the enjoyment of one person may be reliant on that of another. When eating out at a restaurant, for example, people's behaviors might be adapted so as not to detract from the enjoyment of others (Warde and Martens, 2000). Enjoyment of the food can therefore be more than just sensory pleasure, but also social pleasure of enjoying food in the company of others. There are rituals that might be followed to indicate pleasure—noises of satisfaction to show one's appreciation (such as burping) or words of appreciation—when eating food provided by another (Visser, 2017). One person's enjoyment is dependent on enjoyment for all (Warde and Martens, 2000). Examining “enjoyment” is often done through questionnaire or interviews after the meal, identifying broad patterns but relying on parental accounts of the meal (e.g., Skafida, 2013). As with psychological literature, the focus in sociological and anthropological studies has tended to rest on “taste” as either personal preferences or cultural capital. Ochs et al. (1996) on socializing taste, for instance, noted cultural differences between Italian and American families in terms of conversations about the enjoyable aspects of eating. While the synaesthetic and social aspects of taste have been argued (Korsmeyer and Sutton, 2011), there is a lack of research that examines moments of tasting in mundane social settings. The physiological aspects of eating—the visceral processes of ingestion, for instance—have been, until quite recently, largely avoided (Warde, 2016).

### On Prioritizing Health Over Pleasure

The limited research focus on eating enjoyment may be due, in part, to the prevailing concern across the social and behavioral sciences with food and health. Given that our eating practices are one of the primary influences on our health and wellbeing, this is perhaps not surprising. The problem, however, is that the pleasures of eating have typically been positioned as opposing health, sometimes referred to as the asceticism vs. consumption dialectic, in resisting, or embracing the pleasures of eating (Lupton, 1996). Discourses of health have often been contrasted with indulgence, in that to control one's eating is contrasted with eating for pleasure (Warde, 1997). The coupling of abstinence and food has a long history: early Christianity was caught between norms around sharing of food, while also controlling the types and amounts of food to be eaten (Coveney, 2014). Enjoying one's food has thus been overshadowed by principles of civilizing appetites (Mennell, 1987) and controlling bodies (Ogden, 2010). In food advertising, health and enjoyment are even treated as mutually exclusive categories, with foods being targeted as either what parents want (healthy food) compared to what children want (enjoyable food; Burridge, 2009).

This dichotomy has further propounded the notion of eating enjoyment as primarily an individual characteristic, as a pleasurable sensory or experiential state of being, rather than as something that might be shared together. Similarly, prevailing discourses of health often foreground individual responsibilities, control and abstinence (Vogel and Mol, 2014). Whether eating food for health and for pleasure, both have typically been characterized within a psychological, individual framework. To enjoy food is thus to embrace the sensory pleasures of food. As noted earlier, this is too close to sexual pleasure for some researchers: the senses of taste, smell, and touch have been marginalized compared to those perception or hearing, at least within psychology (Eccleston, 2016). Even within the literature on food and health, the pleasures of food have been under-explored. As noted by Coveney and Bunton (2003, p. 162), “pleasure lurks in the background of western thought like a ghostly shadow; neither fully present nor fully absent.”

There are some, however, who are beginning to challenge this constructed division between health and pleasure (Mol, 2012; Cornil and Chandon, 2016). The moralistic undercurrent that runs through this dichotomy is explored in Vogel and Mol (2014) account of dieting advice in the Netherlands, in which a small group of dieticians are promoting self-care and mindfulness (is this food good for me?) rather than restraint and punishment (am I being good?). Focusing on the sensory pleasures of food was also found, experimentally, to lead people to eat less while enjoying the food more (Cornil and Chandon, 2016). What is becoming clear, therefore, is that eating enjoyment has been a neglected research area across the social sciences, characterized primarily in terms of individual pleasure and marginalized in favor of health. These patterns continue as we focus more closely now on how eating enjoyment has been considered within children's eating practices.

### On Children's Enjoyment of Eating

That children might enjoy food, and that this enjoyment might be crucial to understanding their eating practices, has long been evidenced in the child feeding literature (e.g., Cooke et al., 2003; van der Horst, 2012). In this research area, however, enjoyment has rarely been examined as a concept *per se* (Marty et al., 2018). It has instead been treated as synonymous with food preference, a psychological concept that has been more strongly associated with individual traits and measured through children's “liking” of food (see also Mela, 2006, for discussion of the blurring of these concepts).

It is worth considering how enjoyment is typically measured in this field, since these methodological practices highlight the focus on the individualistic aspects of enjoyment. The literature in this area has to date relied heavily on parental responses, through either quantified scores (questionnaires) or verbal accounts (interviews), occasionally supplemented with video observations. In the widely used Child Eating Behavior Questionnaire (CEBQ, Wardle et al., 2001), for instance, children's “enjoyment of food” is scored through parental responses to the following four questionnaire items: “my child loves food,” “my child is interested in food,” “my child looks forward to mealtimes,” and “my child enjoys eating.” Each of these items is rated according to the following options: never, rarely, sometimes, often, always. Research using the CEBQ has tended to show that higher rates of eating enjoyment are correlated with eating more, and a greater variety of foods (van der Horst, 2012). If parents use rewards, persuasion, or pressure to eat, then enjoyment is likely to be reduced (Finnane et al., 2017). Even with infants, the CEBQ was used to demonstrate that there is little difference in enjoyment of food regardless of whether spoon-feeding or baby-led weaning is used (Brown and Lee, 2015).

The focus on parental responses has been for good reason, since it is parents who are largely in control of their children's feeding, particularly in the early years. Infant feeding research has begun to make greater use of video recordings and observations to examine parents' responses to infant gestures (Hetherington, 2017). This work has been important in highlighting the social and interactional aspects of feeding children, and of the subtle cues in facial gestures that are used by parents to determine their child's eating practices, particularly during complementary feeding of young infants (Hetherington et al., 2016). In examining children's facial expressions separately to parental responses, enjoyment of a food is conceptualized primarily as individualistic, such as a biophysiological, cognitive or experiential event. Much of the research in infant feeding therefore seeks to gain objective measures of enjoyment. This is why in some studies of observed infant feeding, parents are asked to wear a mask over their mouth and refrain from talking, so that infants' expressions “were a reflection of their hedonic responses to the food rather than imitation of their mother's facial expressions” (Forestell and Mennella, 2012, p. 1139).

One could conclude, therefore, that the infant feeding research typically examines children's enjoyment of food by asking parents retrospectively whether, and how much, they think their children enjoy their meals. There are a number of concerns with this. First, it focuses attention on parental assessment rather than children's assessment and assumes that another person can make an accurate judgment of this on the basis of a self-report questionnaire item. Second, it treats enjoyment as an overall assessment of “typical” behavior at mealtimes; the contextual specifics of particular meals or foods are thus lost. Third, it is open to response bias as to expectations that meals should be enjoyable (parents may thus respond more positively). Fourth, as with many questionnaire formats around feeding, there is no option for participants to expand on their responses and to provide details as to *what it is* that makes the meal enjoyable, nor how or when the enjoyment becomes relevant.

### On Enjoyment as an Interactional Practice Within Infant Mealtimes

To summarize, there is a paucity of research on eating enjoyment across the social sciences, and even less that focuses on enjoyment as a social practice. With regards to children, very little is known about how the pleasures of food become part of their eating practices. The current paper therefore examines the earliest moments of infant feeding to contribute to this area and to help bridge the gap between cultural and psychological research on eating enjoyment. The analysis also has relevance for conversation analytic and developmental psychology work on caregiver-infant interaction, particularly during weaning, and for emerging work on sensory practices in interaction.

As noted above, video observational work on infant-feeding interactions has received limited attention to date. A few notable studies of mother-infant dyads have begun to detail the mechanics of weaning in terms of the embodied coordination of parent and child (Negayama, 1993; van Dijk et al., 2012; Toyama, 2013, 2014; Costantini et al., 2018). These studies note the fluctuations of feeding interactions and of the increasing coordination of mothers' arm movements and infants' mouth movements. Drawing on the concept of synchrony in caregiver-infant behavior, clear patterns in non-verbal behavior were noted (Costantini et al., 2018). For instance, mothers often opened their own mouths in eating-like movements just at the moment when infants themselves were eating (Negayama, 1993; Toyama, 2013). As weaning progressed, infants opened their mouths before the spoon approached, and the fluidity of spoon-to-mouth-and-removed increased (van Dijk et al., 2012; Toyama, 2014). Other observational research on infant feeding has also begun to examine the role of infants' eye gaze in the coordination of feeding (Kochukhova and Gredebäck, 2010) and indicators of hunger or satiety (McNally et al., 2019). The current paper adds to this collection by examining the verbal (specifically, non-lexical sounds) of the parents alongside the embodied movements of hands, spoons, and food.

Eating enjoyment, considered here as an interactional practice, can also be understood as part of a range of embodied behaviors that are intersubjective and observable phenomena (Majid and Levinson, 2011) through the ways in which they are interactionally organized (Mondada, 2018). In this way, the paper aims to contribute to emerging linguistics work on “sensory practices,” rather than senses *per se* (e.g., Guth and Runte, 2017; Mann, 2018). Previous discursive work on food pleasure has begun to examine how the enjoyment of food can be understood theoretically as an interactional achievement; something that is worked-up and collaboratively produced in talk rather than an automatic process (Wiggins, 2002; Sneijder and te Molder, 2006).

The analysis in this paper focuses on the occurrence of non-lexical vocalizations during the weaning process and thus also contributes to work on sound objects in everyday conversational English (Reber, 2012). Specifically, it is the gustatory *mmm* that is of interest, distinguished by its extended and emphasized form, typically lasting longer than a continuer or other form of “mm” in conversation and as accompanying eating and/or drinking episodes. In earlier work on this (Wiggins, 2002), only audio recordings were used and no attention was paid to the distinction between who was uttering the *mmm*, nor where this was placed sequentially within the meal. The current work also specifically examines the gustatory *mmm* in the context of caregiver-infant interaction during mealtimes. As Mondada (2009) has noted, food evaluations may appear at certain moments: when food is offered, when there is a closing down of a topic, and at “delicate” points in which conflict may be occurring. Just as it is overly simplistic to assume that parents' questionnaire ratings can provide an accurate account of infants' presumed enjoyment of food, so is it also simplistic to equate the gustatory *mmm* with an enjoyable experience. It is important to stress, therefore, that this is not the point. The gustatory *mmm* is not being used as a shorthand indicator of a putative internal state. It is, by contrast, examined in terms of how it *enacts eating as enjoyable* at specific points in mealtime interaction. That is, that the food is *oriented to* as something that can be enjoyed and that this is produced as an observable and socially-relevant object in interaction.

The aims of this paper are therefore to examine where, when and how the parental gustatory *mmm*- as an embodied non-lexical vocalization that orients to food as being enjoyable—is produced during mealtimes with infants between 5 and 8 months old.

## Methods

### Data and Participants

The data comprises video recordings from five families living in Scotland, who recorded the occasions in which they fed their infants over a period of 2–3 weeks during the summer of 2014; these are referred to as the “infant meals.” Participating families were recruited via a short advertisement on a university online noticeboard; families were either university staff or students, or who had heard about the study through university colleagues. There was no payment for participation, though each family was provided with a DVD containing short clips from their recorded meals. Each family was provided with two small video cameras, memory cards and tripods, and instructed on how to set up the cameras so that both the infants' and parental faces could be captured simultaneously. One family (#5) requested to use their own mobile devices to record their meals, and these were typically very short, often just a few minutes of spoon-feeding their infant a snack while seated in a baby walker. All parents were asked to record as many of their infant's meals as possible during the recording period, to become accustomed to the video camera and to collect a variety of meals (e.g., different times of the day). The only demographic information collected about the families were the ages of the parents and the infant. As far as the researcher was aware, the infants had no clinical feeding problems or dietary restrictions.

### Coding and Analytical Procedure

Across the five families, 66 meals were recorded, with a total of almost 19 h of video data from infant meals. Families #1 and #2 (see Table 1) used baby-led weaning, in which their infants were more autonomous in their feeding and provided with small pieces of food rather than a solely spoon-fed diet. Following data collection, the full set of video recordings were searched manually for all and any references to enjoyment of food, whether through lexical (“did you enjoy that”) or non-lexical (“*mmm*”) embodied sounds. As noted previously, an orientation to enjoyment can be made through various means, such as references to the food being “yummy,” gustatory *mmm*s, lip smacks, or other non-lexical sounds such as “ooh,” “ah,” or an audible in- or out-breath with pursed lips (similar to an “ooh” but as a breathy sound rather than a vocalization). When interacting with infants and small children, the onomatopoeic sound “nom-nom” might also be used. It is important to note that although the infants are able to produce non-lexical sounds themselves, this study focuses on parental use of gustatory *mmm*s as orientations toward enjoyment or pleasure. Coding of the data was undertaken by the researcher alone, with each instance of a lexical or non-lexical orientation to enjoyment noted in terms of its form (e.g., “*mmm*,” “*ooh*,” or lip smacks) and timepoint within each meal. The coding was conducted manually, through careful viewing of all video-recordings within the corpus, and inclusive, noting borderline cases such as “*mms*” that were not necessarily gustatory. In the interests of analytical focus, however, only the gustatory *mmms* are included in this paper.

**Table 1:** Number of gustatory *mmms* across the data corpus.

**Family**	**Age of infant**	**Meals recorded**	**Total recorded time (h:mins)**	**standalone mmms**	***mmm* + object-side**	***mmm*+ subject-side**	**Total *mmm*s**
#1	7 mths	16	08:39	132	39	1	172
#2	8 mths	14	05:55	18	5	0	23
#3	5 mths	9	01:56	35	6	0	41
#4	5 mths	15	01:35	25	7	0	32
#5	6 mths	12	00:32	5	0	0	5
Totals		66	18:49	215	57	1	273

All sections of the data which featured instances of the parental gustatory *mmm* were then identified and transcribed, including the sequential turns immediately prior to, and following, the *mmm*. A gustatory *mmm* was coded as “standalone” if there was a pause of one second or more between the *mmm* and further assessments, or as “*mmm* + assessment” if there was an assessment token (such as “nice,” “yummy”) immediately after the *mmm*. These sequences were then analyzed using discursive psychology (Edwards and Potter, 1992; Wiggins, 2017), an analytical approach that examines how psychological concepts (such as enjoyment) are discursively constructed and used in social interaction. The analysis focused on the form of the *mmm*s, where they were positioned in the meal and in relation to non-verbal, embodied practices (such as handling spoons, chewing food or using hands to pick up objects). It is important to distinguish this kind of analysis from other ways of coding feeding practices, which focus on categorizing parental behavior into “prompts,” for example. By contrast, discursive psychology focuses on the interaction between parent and child, and examines talk not only in terms of its sequential and contextual placement, but also through participants' rather than analysts' orientations. This means that the gustatory *mmm*s were not treated as a uniform category of, for example, modeling, or prompts to eat food, but instead were examined in terms of how they were used or oriented to by participants.

### Ethics

Working with data involving small children and video recordings from family homes clearly generates ethical issues, particularly around consent, and the use of data extracts. Ethical approval was first acquired from the University of Strathclyde ethics committee before embarking on the research. Participating families were then recruited through posters and a university emailing list, with a particular focus on those families who were weaning their first child. All prospective parents were then contacted and met in person to discuss the study, and full written consent was obtained from all parents involved. Moreover, parents had full control over the video cameras and recordings; they alone set up the cameras (the researcher never visited the participant homes), took the recordings, and had the opportunity to review and delete any recordings that they did not wish to be used. Parents also gave consent to use anonymized still images or video clips for academic publications and presentations.

## Results

The format and sequential positioning of gustatory *mmms* in the infant meals was found, in most cases, to follow a clear pattern, indicating specific moments at which parents oriented to enjoyment of the food or meal. In particular, just over half of the *mmms* occurred at the precise point in which the infant's mouth closed around a spoonful or handful of food, thereby situating enjoyment as an immediate embodied and gustatory experience. The results section will first overview the number and format of *mmms* across the five families before detailing the sequential positioning and the construction of enjoyment. Table 1 specifies the number and format of *mmms* identified across the full corpus, with details of how many were identified for each of the five families.

A few points are worth highlighting here. First, as noted earlier, families #1 and #2 used a baby-led weaning approach, which meant that they often ate their own meals alongside their child or else supervised the infants' self-feeding while doing other activities nearby (e.g., cleaning or tidying the kitchen). Although no conclusions can be made on the basis of two families, the difference in the overall time taken for meals is notable; when infants fed themselves, the meals lasted much longer. Second, all but one of the *mmms* was used in the context of eating or orienting to food; the odd one out was produced when the infant was drinking water. Third, all of the *mmms* followed a similar prosodic pattern, with an emphasized and prolonged “mm” sound, sometimes with rising or falling intonation (or both), and all uttered with a closed mouth; in some instances, the sound was elongated or exaggerated.

Confirming a pattern noted in previous research (Wiggins, 2002), the gustatory *mmm*s were overwhelmingly “standalone” (215 out of 273 instances, around 80%), uttered in first position without any preceding or following lexical item, or clarification regarding the role or purpose of the *mmm*. As such, they were characterized by spontaneity, immediacy, and vagueness: they could be spontaneously produced without any prefacing or pre-announcement, were located immediately at the start of a turn in talk, and were typically unaccompanied without any explanation about the source of the enjoyment. Unlike previous research on *mmms*, however, the analysis considered the distinction between object-side and subject-side assessments (Edwards and Potter, 2017), and as can be seen from the table there is almost an exclusive presence of object-side assessments. That enjoyment is often conflated specifically with “liking” in the child feeding literature is therefore of concern. While the lack of subject-side assessments does not mean that *mmms* could not indicate an infant's liking of a food, the findings here suggest that something else is going on with regards to *mmms* and orienting to enjoyment that relates more to the assessment of the food than to personal preferences.

The table above provides an overview of the number and form of *mmms* but no sense of how they were situated within infant meals nor what their purpose or consequences might follow. To investigate further, therefore, the *mmms* were examined in terms of how and where they were sequentially positioned within the meals. Four different types of gustatory *mmms* were noted and were classified in the following way:

1. Announcement: at the introduction of a food to be eaten imminently
2. Receipting: as the food is placed within the infant's mouth
3. Modeling: as the parents enact their own enjoyment of food
4. Encouragement: as infant food consumption slows or is distracted

Table 2 presents the distribution of the four types of gustatory *mmms* across the corpus.

**Table 2:** Classification of gustatory *mmms* across the data corpus.

**Family**	**Announcement**		**Receipting**		**Modeling**		**Encouragement**		**Total**
	***Mmm***	***Mmm + eval*.**	***Mmm***	***Mmm + eval*.**	***Mmm***	***Mmm + eval*.**	***Mmm***	***Mmm + eval*.**	
#1	14	1	71	23	0	1	47	15	172
#2	4	0	7	1	6	3	1	1	23
#3	0	0	16	3	11	0	8	3	41
#4	4	3	21	4	0	0	0	0	32
#5	0	0	5	0	0	0	0	0	5
	22	4	120	31	17	4	56	19	
Total	26		151		21		75		273

The four types of gustatory *mmm* are distinguished in terms of their immediate contextual features rather than their form; there are some differences in prosody and duration of type three and four *mmm*s but otherwise they are fairly consistent. They have been presented in this order, rather than the most prevalent first, since the order mirrors the relative placement within a meal (from the introduction of food, to first taste, to consumption). Each of these *mmm* contexts will now be discussed and illustrated in turn.

### Announcement *mmm*

The first location of a gustatory *mmm* occurs at a point in which a food is first introduced or announced to the infant. These typically occurred in the data corpus at the beginning of the meal, but could also be situated during the meal, when a new food item was introduced. In some cases, the announcement *mmm* was used in the presence of food-related accompaniments, such as bowls, or when putting on the infants' bib or strapping them into their high-chair. These food announcement *mmms* are similar to, but more immediate than, other types of food assessments produced when food is offered at the table (cf. Mondada, 2009). The characteristic feature of these *mmm*s can be summarized as follows: (a) they occur at the introduction of a to-be-consumed food item or at the very start of the meal when the infant is being “prepared” for feeding, (b) the parents' eye gaze is on the food at the moment of utterance, (c) the parent is typically holding the food as the *mmm* is uttered. Extracts 1 and 2 below illustrate this form of gustatory *mmm*.

Extract 1: family #4, Chris (meal 2)^1^

**Table:** 

1.	Mum:	you got ↑that spoon (0.2) I got
		↑this spoon
2.		(5.0)*((picks up bowl, stirs food))*
3.		mm↑m::m. *#figure 1*
4.		(2.0)*((lifts spoon out of bowl))*

Extract 2: family #2, Jess (meal 3)

**Table:** 

1.		(5.0)((*Mum moves packet from table*
2.		*to in front of her and infant,*
3.		*#figure 2*))
4.	Mum:	[mm- mm- ↑mmmm: (0.6).hh mango:
5.		[*((eye gaze flicks up to infant))*

**Figure 1:**
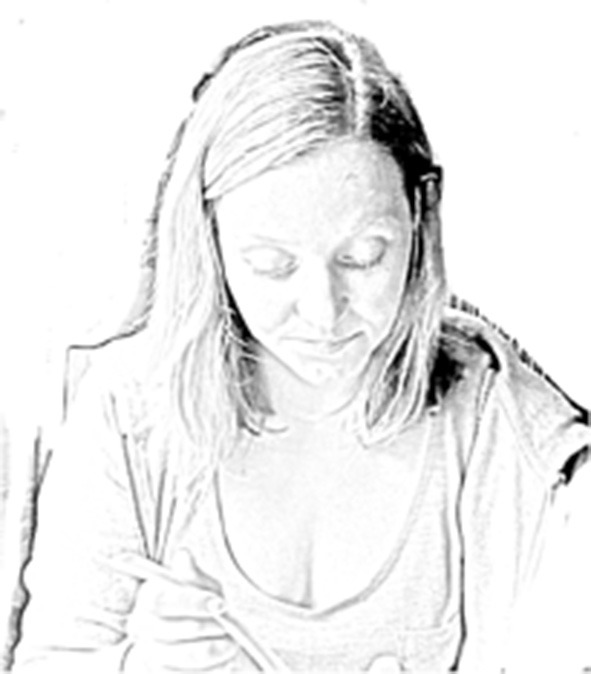
Image published with the written informed consent of the depicted adult.

**Figure 2:**
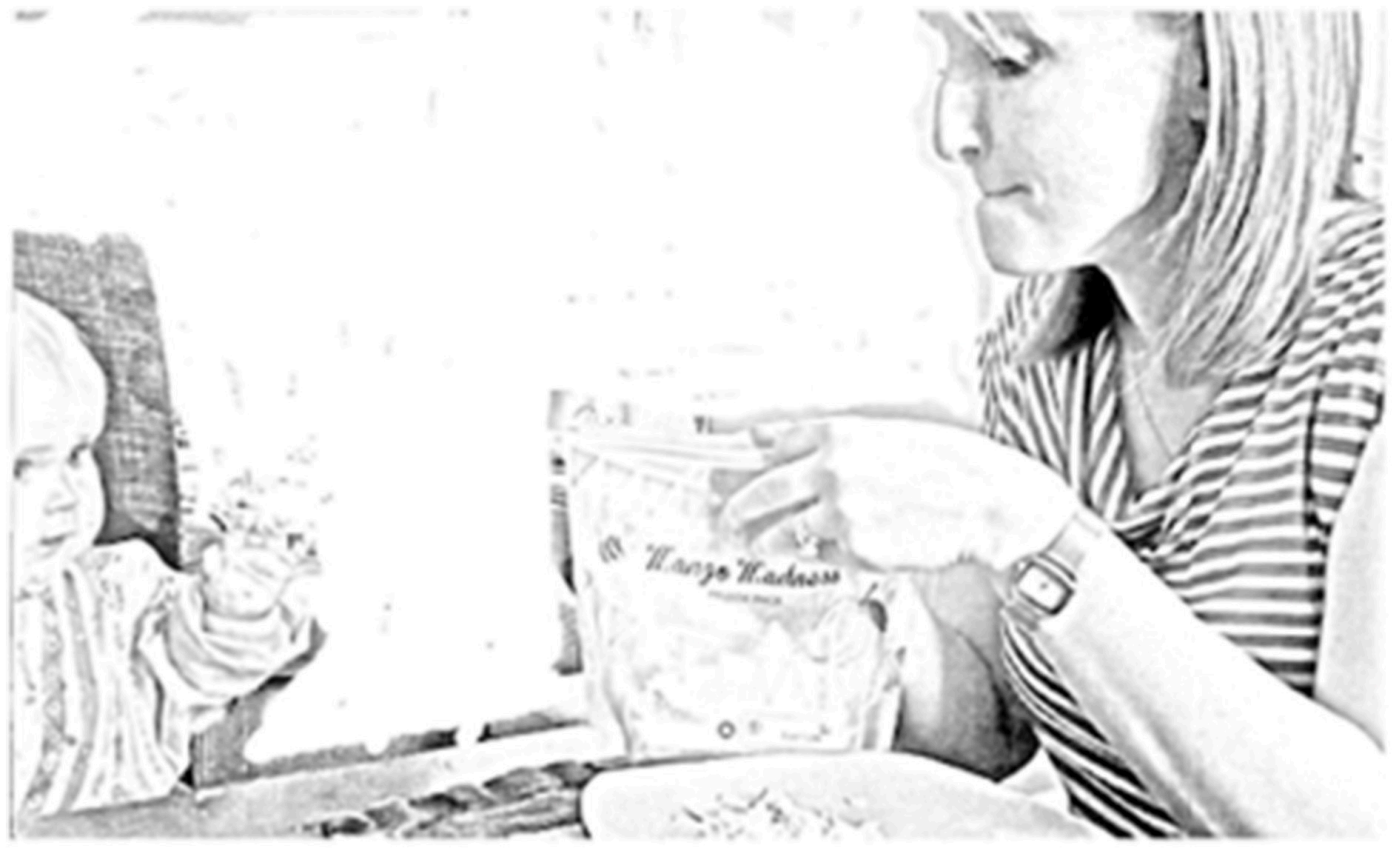
Image published with the written informed consent of the depicted adult and of the parents of the depicted child.

In both extracts above, the *mmms* are preceded by a long pause in which an embodied sequence plays out. The parent picks up a bowl or packet with the anticipated food item inside, sometimes also stirring the food with a spoon (Extract 1, see Figure 1) or opening a packet (Extract 2, see Figure 2). In contrast to most other *mmms* (where parental eye gaze is almost always on the infant), the parental eye gaze during these gustatory *mmms* was partially or fully on the food item. In extract 2, Mum's eye gaze flicks from the food item, to the infant, and then back to the food item. In doing so, she uses gaze both to orient to the food item and to invite the infant to follow her gaze.

There might have been other lexical or non-lexical terms that parents could use at this moment. There are, for example, instances in the data corpus when an audible and extended in-breath (almost, but not quite, an “ooh”) is used to announce a new food, but these typically occur when it is someone else who brings the food. As such, we might speculate that such audible in-breaths enact surprise rather than enjoyment *per se*. By contrast, the prosodic formation of the gustatory *mmm* signals the arrival of the food as being a specific type of object (one that anticipates enjoyment) or of the preparation of the meal as a preface to the enjoyable event.

The sequential positioning of these gustatory *mmm*s is also important here, since in many cases, the food was already present near the parent (therefore the sight and smell of the food might have been noticed earlier) and is only at this moment being brought to the infant's attention as a relevant food item. The *mmm*s then “announce” the food as next on the menu, and sometimes (as in extract 2), the name of the food is also tagged on. While no explicit assessment of the food has been given (e.g., “this mango will taste nice”), the gustatory *mmm* does the work of orienting to enjoyable qualities of the food without having to specify what exactly those qualities might be. What is important, instead, is that the food is enacted as anticipating enjoyment *at just this point in the interaction* and thus serves to foreground the relevance of the food for the infant immediately prior to eating.

### Receipting *mmm*

The most common sequential position—accounting for around half of all gustatory *mmms* in the corpus—was located at the exact point in which food had been taken into the mouth, either by spoon or hands, and with visible mouth closing or jaw movements. These gustatory *mmms* were often uttered at predictable moments, not with every mouthful of the infant, but at a recognizable point at which the taste of the food might be said to have been “received.” As such, I refer to them as the receipting gustatory *mmm*, since they focus attention on the moment at which a taste experience might observably have begun (when the food is placed within a closing mouth) rather than on the eating process *per se*.

The characteristic features of these *mmm*s were as follows: (a) they were uttered temporally when the mouth closed round the spoon or the spoon was withdrawn from the mouth, or as the child's hand with food went into the mouth, (b) parental eye gaze was always on the child's face, (c) typically following a pause or verbal silence, (d) were usually standalone *mmms*. These *mmm*s occurred in the same sequential location regardless of the feeding approach, whether the parents were spoon-feeding or the infant was feeding themselves with hands or a spoon. Extracts 3 to 6 below detail this pattern; images have been used where possible to illustrate the co-ordination of hands, food and mouths.

Extract 3: family #4, Chris (meal 08)

**Table:** 

1.	Mum:	Mummy talking nonsense again
2.		(3.2)*((spoon into mouth))*
3.	Mum:	mm↑mm:, *((figure 3))*
4.		(1.2)*((spoon withdrawn))*
5.	Mum:	is that ↑ nice

**Figure 3:**
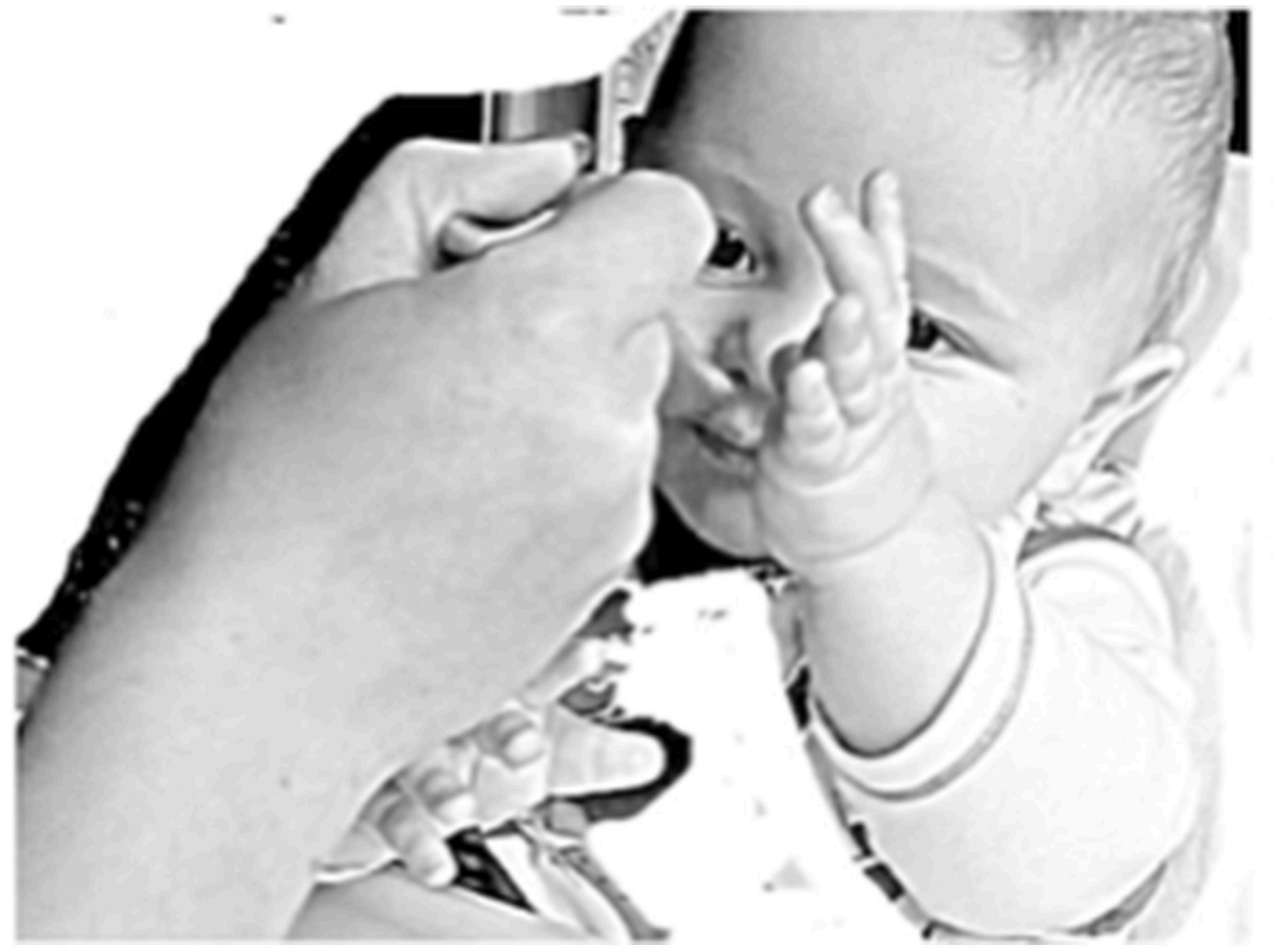
Image published with the written informed consent of the parents of the depicted child.

In this family, the parents used spoon-feeding, and as such the lengthy pause (line 2) is due to the time taken to guide the spoon toward the mouth and to ensure that the infant opens their mouth at the right point in which to allow the spoon to enter (cf. Toyama, 2014). Interestingly, the same silence before the *mmm* often occurs even in those instances in the data corpus when the infant is feeding themselves, while the parent watches the food being lifted up into the infant's mouth. Interactionally, this auditory silence allows for a break in any talking and enables the focus to rest on physical manipulation of the food. The *mmm* then occurs as a turn-initial sound for the parent, though we might also treat it as the second part of a paired action, with the food placed on the tongue as the first pair-part. Indeed, it could even be a third part, with the following sequence: (1) food into mouth, (2) mouth closed around food, (3) *mmm* as receipt of the taste (see Figure 3). As such, the infant's embodied movements (closing of the mouth around the food) might be treated as a grammatical turn (Keevallik, 2018), with the tasting of the food as much a part of the interaction as the verbal utterances (Mondada, 2018).

The next extract (4) below illustrates how it is the precise moment of food going *into* the mouth and being accountably “received” by the infant that provides the crucial part of the timing of the *mmm*. In this extract, Mum has been spoon-feeding 6-month-old Lucy, who is sitting in her baby walker (a chair with tray and wheels), and as such needs to negotiate the movements of mouth, spoon, and infant.

Extract 4: Family #5, Lucy (meal 10)

**Table:** 

1.	Mum:	here comes the↑airplane = whoosh::
2.		(3.0) *((spoon moved toward mouth,*
		infant moves))
3.		*((#figure 4, image 1+2))*
4.	Mum:	mm↑mm, *((#figure 4, image 3))*
5.		(3.0) *((spoon retracted))*

**Figure 4:**
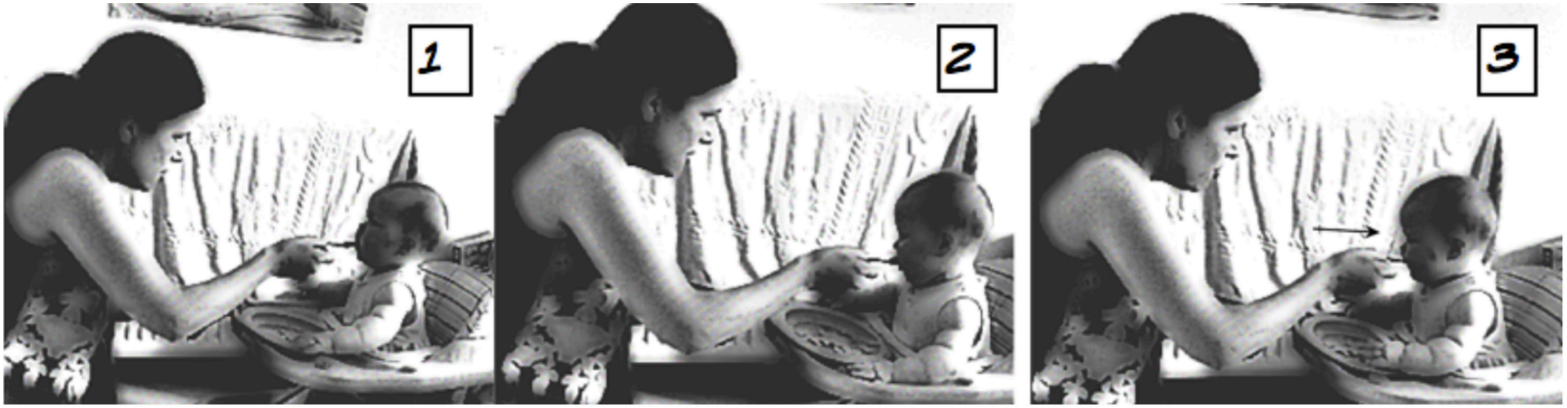
Image published with the written informed consent of the depicted adult and of the parents of the depicted child.

In this example, the silence immediately preceding the *mmm* is punctuated with two attempts by Mum to get the food into Lucy's mouth; see images 1 and 2 (Figure 4). In the first attempt, Lucy is looking up toward her Mum but the spoon does not go into the mouth and Lucy's head turns away. In the second attempt, the spoon again touches her lip but Lucy's head moves before the food goes in. It is only on the third attempt that the spoon enters the mouth, and in a swift retracting movement Mum removes the spoon while uttering the *mmm* (lines 3 and 4). As with the other examples, the timing of the *mmm* is crucial here, since it points to the closure of the mouth around the food—and thus “a successful attempt”—rather than the taste of the food on the lips or other parts of the mouth.

There is evidence that the *mmm* works as much as an assessment term on its own (in reference to something being “good” or “nice”) as much as it does a marker of enjoyment or pleasure in particular (see Wiggins, 2002). In some cases, such as extract 5 below, parents make explicit their orientation to checking their infant's assessment of the food. In this example, Jess has been eating for some time; her parents have finished their own meal and it is Dad who stays to sit with Jess and talk to her as she continues to eat. Jess is picking up and chewing food on her own with no assistance of spoons, nor does Dad pick up any pieces of food for her. This example is an illustration of how the method of eating (in this case, baby-led weaning) did not make any difference with regards to the sequential organization of either announcement or receipting gustatory *mmms*.

Extract 5: family #2, Jess (meal 1)

**Table:** 

1.	Dad:	[they are good aren't they
2.	Jess:	[*((picks up food))*
3.		(0.4)*((food into mouth))*
4.	Dad:	mmmm, *((figure 5))*
5.		(2.0)
6.	Dad:	they're yummy

**Figure 5:**
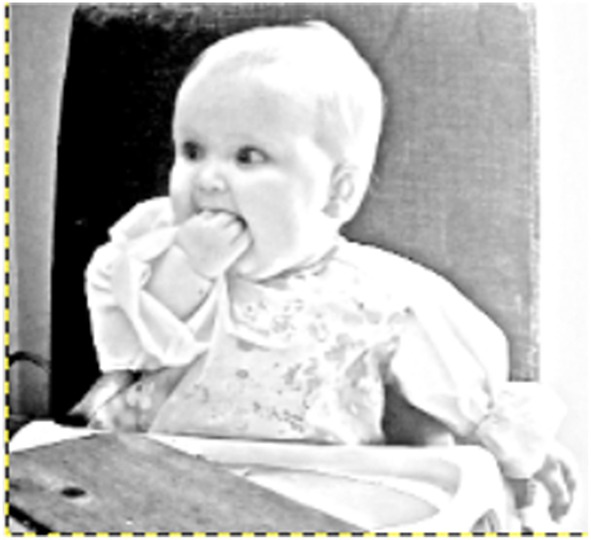
Image published with the written informed consent of the parents of the depicted child.

In this extract, Dad orients to Jess's continued eating as confirmation that the food is “good” and “yummy” (lines 1 and 6). As Jess picks up more pieces of food, for instance, Dad's assessments are in overlap. The receipting *mmm* then occurs as Jess's hand (with food inside) is placed into her mouth; at this point her eye gaze is directly on Dad (Figure 5). As before, the *mmm* takes place in third position: food picked up -> mouth closes around food -> gustatory *mmm*. Dad's explicit assessment “they're yummy” (line 6) then works to confirm the assessment verbally. In contrast to extract 3—in which the parent did an assessment check—here the assessment builds on the *mmm*. The various possible combinations of *mmm*s and lexical assessment terms therefore suggests that the *mmm* functions as both complementary to assessments but also adding something qualitatively different.

The final example for the receipting *mmm* illustrates how it can be repeated soon after the first utterance. While the *mmm* is predominately uttered without any other assessments or lexical terms, in this example each *mmm* is of the form “*mmm* + object-side assessment.” This family uses baby-led weaning and at this point in the meal Mum has just passed a rice cracker to 7-month-old Sarah who then puts into her mouth.

Extract 6: family #1, Sarah (meal 3)

**Table:** 

1.		(2.0)*((Sarah bites the cracker))*
2.		(5.0)*((cracker out of mouth, then*
3.		*back in again))*
4.	Mum:	°mmmm: ° (0.2) >nice<*((Figure 6,*
		*image 1))*
5.		(4.0)*((Sarah looks at Mum,*
6.		*food out of mouth))*
7.	Mum:	mm↑mm: (.) ↑yummy *((image 2))*

**Figure 6:**
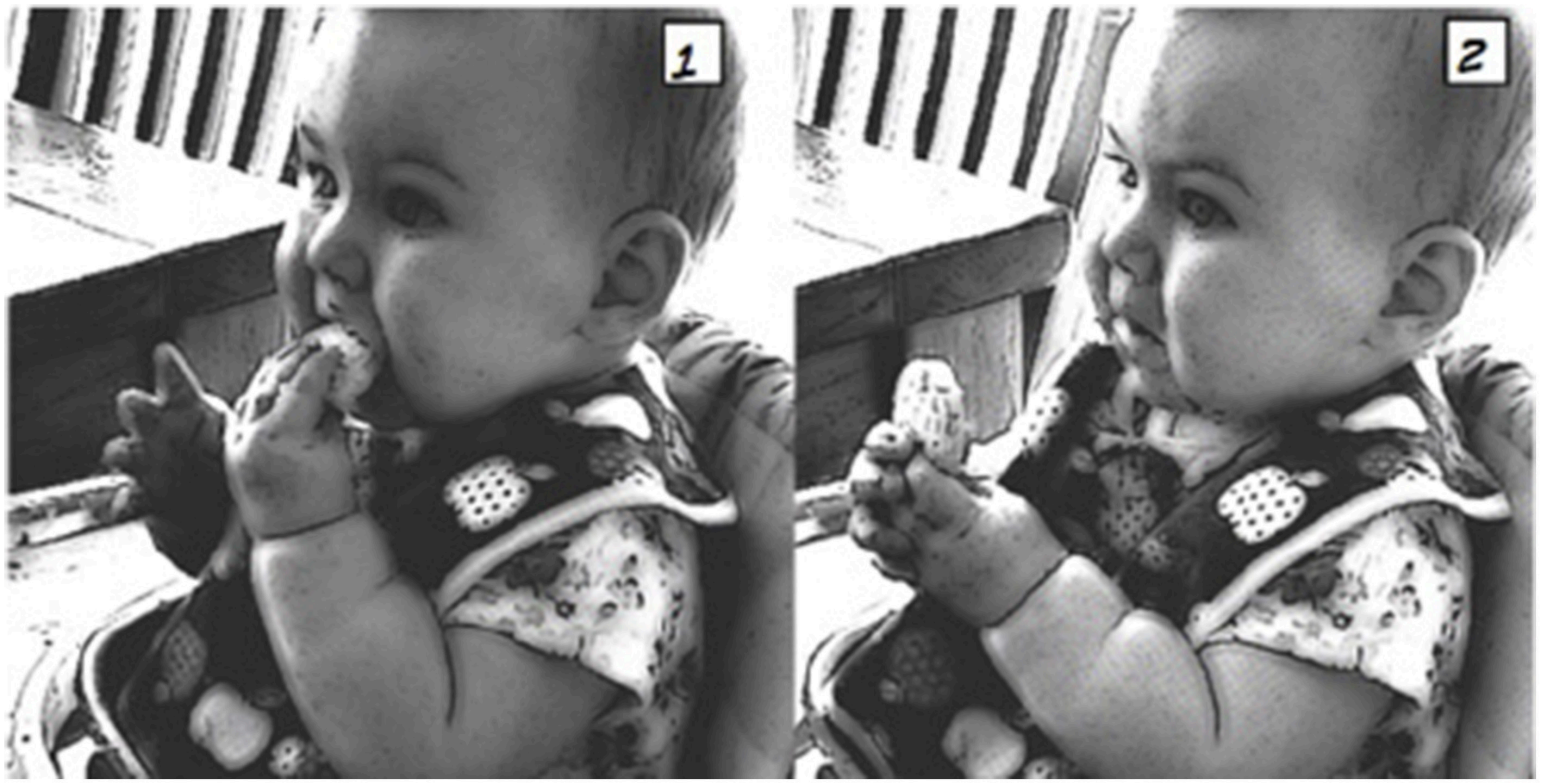
Image published with the written informed consent of the parents of the depicted child.

The recycling of the *mmm* can be seen to occur at just the point at which Sarah looks toward her Mum (see Figure 6). In extract 6, both *mmms* are of the form “mmm + object-side assessment” (“nice,” “yummy”) and thus do a little extra work to specify the focus of the utterance. The second occurrence of the *mmm* has a slightly rising intonation, with an almost confirmatory tone. What is important here is how they work to bind together the non-lexical *mmm* with the lexical and positively-loaded assessment particles. While the presence of standalone *mmms* is evidence that they work sufficiently well without an assessment term, the occurrence of the *mmm*+object-side assessment provides confirmation that the *mmm* is itself positively loaded.

It is worth reiterating the point that, in most cases, the parents are not eating any food themselves while producing these *mmms*. They are, then, an utterance produced as an explicit orientation to the assumed gustatory experiences of another person (their child). To do so at just the moment at which a mouth closes around a spoon, or a piece of food, illustrates a practice that was observed across all five families, repeatedly, and with different constellations of food, hands, and utensils. Such an observable pattern is remarkable: not only that different parents produce an utterance that attends to their infant's consumption at such a specific time-point, but also that it is produced in such a common way with similar intonation, sequentiality, and eye gaze. In the 7 out of 143 instances which involved the parents tasting the food, the pattern remained the same: the *mmm* was located just as the mouth closed around the food, and eye gaze from parent was fixed on the child.

What is particularly noteworthy about these *mmms* was that parents always began the utterance when looking directly at their child's face, even if their gaze moved before they had finished uttering the *mmm*. This contrasts sharply with a study on tasting between strangers, in which mutual eye gaze between cheese shop owners and customers are avoided during the moments of tasting a piece of cheese (Mondada, 2018). In this sense, an “individual space” is created for the taster and an orientation to tasting as being something different from eating. As Mondada notes (2018, p. 754), “tasting is not a private experience, but an individual experience that has a public, witnessable, accountable, and intersubjective dimension.” By contrast, the gustatory *mmms* found in this corpus suggest an orientation to a particular positive assessment—enjoyment—rather than opening up the interaction for a response from the infant^2^.

### Modeling *mmms*

The third type of gustatory *mmms* occurred when parents were eating food and during which a more explicit modeling of eating enjoyment was enacted. The occasions occurred when the meal was underway and when parents were eating themselves, usually their own food but on some occasions a food that was being eaten by the infants themselves. These were less predictable in terms of their sequential placement within a mealtime, or within the feeding of the infant, but they nevertheless had the following core features: (a) were uttered when the parent themselves were eating, (b) parental eye gaze was on the child, (c) were slightly exaggerated or extended *mmm*, often comprising several *mmms* together or a combination of *mmm* plus another lexical or non-lexical marker (e.g., lip smacks, or “nom nom”). In some cases, the parents closed their eyes during the production of the *mmm*—despite having started with eye contact with the infant—and this further enabled an enactment of individual pleasure. As with many of the other *mmms*, they followed an extended pause during which the parent was eating. Extract 7 illustrates the ways in which these modeling *mmm*s often comprised multiple *mmm* components; in this meal, Mum is eating her own breakfast while seated opposite Daisy, who has been eating for some time and is continuing to pick up small pieces of food herself:

Extract 7: family #3, Daisy (meal 8)

**Table:** 

1.		(4.0)*((Mum looks down at food))*
2.		(1.6) ((*Mum looks at Daisy, food*
		*intomouth,starts chewing))*
3.	Mum:	mmm = mmm (0.2) ↑mmm *((nodding,*
		*eye gaze on Daisy))*
4.		(3.0) *((Mum stops chewing,*
		*eye gaze on Daisy*))
5.	Mum:	yummy yummy yummy
6.		(3.0) *((Mum continued eye gaze on*
		*Daisy*))

What distinguishes this *mmm* from a receipting *mmm* is that it occurs not at the point of the mouth closing around the but at the point at which the parent is visibly chewing food: this is eating, rather than tasting, food. Mum's eye gaze is fixed on Daisy from lines 2 to 6, and so the *mmm* is as much directed at Daisy as it might be on Mum's own sensory experiences. The repeated *mmms* (line 3), with slight upward intonation on the final *mmm*, present a more exaggerated and extended form of gustatory *mmm* than seen in either of the first two classifications. In this sense, this third type of *mmm* seems to be doing some work to “model” enjoyment of eating through the parent's own enactment of this while eating their own food. Similarly, the three-part “yummy yummy yummy” (line 5) works to focus attention on the action being performed here as much as the assessment itself. That is, it is the doing of an assessment and its observability—the orientation to food as being “yummy”—that is important here. A single “yummy” might focus attention on the food through making an assessment, whereas a three-part “yummy” focuses attention on the assessment *as a relevant thing to do* at just this point in time.

The modeling of eating enjoyment might not only be considered as a way to role-model a normative practice during eating, it might also serve to encourage or motivate the infant to eat themselves. In other words, through modeling enjoyment, parents could model eating as a relevant practice. In extract 8, Jess has been eating her lunch alongside her parents, but has become agitated, stopped eating, and has begun to make crying noises. Her parents then try different actions to calm Jess and encourage her to continue eating, including Dad's extended vocalizations as he eats some of Jess's food:

Extract 8: family #2, Jess (meal 9)

**Table:** 

1.	Jess:	nn: nn-↑nngh::
2.		(1.0)*((Dad picks up a piece*
3.		*of bread and starts eating))*
4.	Jess:	>nng- ↑nng < ↑↑nngh- (0.2)
5.		↑aoo:: [::ww::m
6.	Dad:	[mm:,
7.		(0.6)
8.	Jess:	aow[:::mmh::::
9.	Dad:	[this is ↑lovely Jess
10.		(1.8)*((holds bread up towardJess))*
11.	Jess:	ahm[m::eh:::mmmh-
12.	Dad:	[mm↑mm
13.		(0.6)
14.	Dad:	mm:mm:mmmm mm::mmm: = that was
15.		delicious (.)°mmm°,
16.		(1.0) #*figure 7*
17.	Dad:	mmm::mmm:mmm:mmm.
18.		(1.0) *((Mum passes a piece*
19.		*to Jess))*
20.	Mum:	want to try one

**Figure 7:**
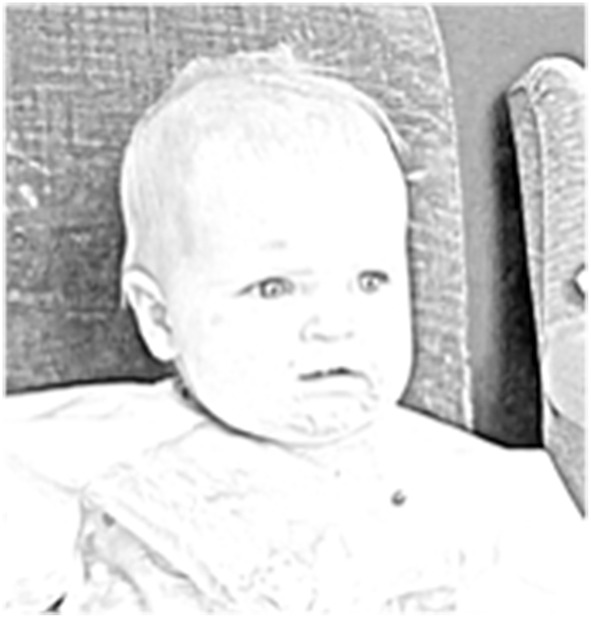
Image published with the written informed consent of the parents of the depicted child.

Dad looks at Jess almost entirely through this sequence, other than for briefly glancing down at the food in his hands. Jess also maintains eye contact (Figure 7) with Dad for the duration of this sequence, having stopped crying around line 12. This rather unusual extended gustatory *mmm* serves to illustrate how it highlights not the food's characteristics but the enactment of enjoyment as being the relevant thing at this point in the interaction. What is key to this sequence is that Dad has visibly taken a piece of bread from the plate of food that is being passed to Jess periodically: he is eating *her* food. The continued eye gaze, raising up of the food to make it more visible, further serve to orient to this apparent transgression. We can also see two *mmm* + evaluation formulations (lines 8 and 13) that further amplify the enactment of enjoyment. That this dramatization by Dad might be a ploy to encourage Jess to eat more is then confirmed by Mum's direct offering of food to Jess in line 17. Following this, Jess does then take the food and continue eating.

In this third type of gustatory *mmm*, then, parents orient directly to their own eating processes—the chewing and taste of food—by making this audibly and visibly relevant to their infants. Through eye contact at the start of the production of the *mmm*, they demonstrate that the *mmm* is a social act: not just an expression of their gustatory pleasure, but an interactionally relevant thing to do.

### Encouragement *mmms*

The fourth type of gustatory *mmm* was produced at various sequential locations within the infant mealtimes, though they typically occurred when the parents also oriented to potential resistance from the child with regards to eating. For instance, when the child looked unsure about the food, spat it out, stopped chewing, or was otherwise distracted by something else. These *mmms* are therefore named “encouragement *mmms*” as they appear to be tied up with a specific social action: to encourage the infant to begin, continue, or resume eating. They had features similar to those seen in the third type (modeling *mmms*), though in this case, the parents were not themselves currently eating any food. Encouragement *mmms* were a more varied category than the previous three but can be distinguished by the following features: (a) either before (as food is being offered) or during infant eating, but typically when infant not actively or visibly chewing, (b) sometimes a more exaggerated or elongated *mmm* or accompanied by other lexical (yummy) or non-lexical (lip smacks) sounds, (c) parent is not chewing food themselves at this point, (d) parental eye gaze on the child, (e) often accompanied by checks with regards to taste or consumption (e.g., “do you not like that?”) or when there is possible resistance to the food.

These categories were most commonly seen in families #1 and #3, at points in which the infant was eating from a spoon (held by themselves or their parents) or else were picking up small pieces of food from a tray. In extract 9, we see an example of how these encouragement *mmm*s might accompany the immediate offer of a food to the infant.

Extract 9: family #1, Sarah (meal 14)

**Table:** 

1.	Mum:	looks like porridge now not
		just watery
2.		(4.0)*((Mum blows on food to*
		*cool it))*
3.		*((spoon put in front of Sarah))*
4.		(2.0)*((Sarah looks up at Mum, no*
		*hand movement*))
5.	Mum:	mm:, (0.2) got strawberries, (.)
		plums in it
6.		(2.0)*((Sarah looks up at Mum,*
		*grasps spoon*))
7.	Mum:	mm↑m
8.		(1.8)*((Sarah looks down and puts*
		*spoon into mouth*))

Prior to this extract, Sarah had been eating pieces of fruit while waiting for the porridge to cool; now the porridge is ready, and Mum presents this to Sarah on a spoon which she usually grasps to feed herself. At line 3, Mum holds the spoon in front of Sarah but there is no immediate uptake (line 4) which might indicate Sarah's lack of readiness to eat the food. The first *mmm* then works here as an encouragement to take (and eat) the porridge. Unlike the announcement *mmm*s, which typically occur as the food is being stirred or attended to before presentation to the infant, this encouragement *mmm* happens as part of the offering of food. It is slightly shorter and less exaggerated in this case—unlike some other encouragement *mmm*s (see extract 11)—but still in initial turn-position and following a brief pause. As such, the *mmm* works more as an assessment of the food to encourage the infant to eat it, rather than anticipation at enjoyment-to-be-had.

That these encouragement *mmm*s work for the most part like an assessment token is further evidenced by extract 10, in which we see an example of an *mmm* alongside an object-side assessment. This is taken from the same family as above but a different mealtime.

Extract 10: family #1, Sarah (meal 10)

**Table:** 

1.	Mum:	you dropped something here didn't
		you look-
2.		*((Mum helps to pick things out of*
		*the highchair*))
3.		(4.6) *((Mum moves away; Sarah*
		*visibly chewing*))
4.	Mum:	mmm::, (.) nice? *((Sarah looks,*
		*to Mum then down*))
5.		(10.0)*((Mum carries on tidying up))*

In this example, Sarah is visibly chewing but there are also pieces of food dropping from her mouth and on her highchair. The *mmm* is then not a response to an announcement of food (announcement mmm) nor immediate taste of a piece of food (receipting mmm), but rather an orientation to an ongoing eating process that Mum herself is not engaged in (cf. modeling mmm). The combined “mmm::, (.) nice” follows a pattern seen in other *mmm* + evaluations in that there is short gap between the non-lexical *mmm* and the lexical “nice.” The gustatory *mmm* in this case becomes more loaded in terms of assessment, though is steered toward an assessment check (with questioning intonation on the “nice,” line 4) rather than an assessment claim by Mum. As with other instances of the *mmms*, this enables the parents to attend to the potential enjoyment of the food without overriding the infants' own abilities to assess the food for themselves. The *mmm* therefore ambiguously orients to the food as being pleasurable without making any claims about the infant's sensory experiences. Had this been a “like it?” subject-side assessment, for instance, then this would position the parents as making assumptions about their child's taste experiences or food preferences (Edwards and Potter, 2017).

In the final example, we see the use of an encouragement *mmm* in a more exaggerated form. On this occasion, Daisy is being distracted by the family cat. Mum makes several attempts to draw Daisy's attention back to the food, and the *mmm* becomes part of this endeavor.

Extract 11: family #3, Daisy (meal 4)

**Table:** 

1.	Mum:	what do you think. (0.4) s'it
		getting the seal
2.		of app↑roval (0.2)>.mpt.mpt.mpt
		.mpt.mpt<
3.		(1.8)
4.	Mum:	^*^>.mpt.mpt.mpt.mpt.mpt.mpt
		< = mmmm^*^m:,
5.		^*^((Daisy looks at Mum))^*^(Daisy turns away)
6.		(.)
7.	Mum:	.h Daisy
8.		(1.4)*((Mum turns to look at the*
		*cat))*

Throughout this sequence, Mum has a spoon held out toward Daisy—with food on it—and Daisy has a little food left in her mouth that she is not visibly chewing. Daisy is focused instead on the antics of the cat, and keeps her gaze on the cat except for a short period (lines 4-5). The “.mpt” here represent a series of lip-smack noises that Mum uses to orient to the food, and specifically, to the eating of the food. The encouragement gustatory *mmm* is placed at the end of the second sequence of lip smacks (line 4) and is accompanied by a smile and an extended prosodic form of the *mmm*. This *mmm* therefore has quite a different sequential organization to the previous encouragement *mmm*s, though the social action within which they are bound up is the same: to keep the child focused on eating the food.

## Discussion

This study has provided a preliminary classification system for four different types of gustatory *mmms* that may be enacted by parents during infant mealtimes, as found in the data corpus from English-speaking families living in Scotland. The classification was based on multimodal features including sequential organization, format and duration of *mmms*, eye gaze, and object (food) manipulation by both parents and infants. It has been argued that these gustatory *mmms* enact and make relevant enjoyment of eating at specific moments in the mealtime, and orient to enjoyment as an interactional and socially normative process around food. Moreover, they appear to orient to different kinds of enjoyment, whether in anticipation of the food (announcement *mmms*) or in relation to the sensory features of the food (receipting *mmms*). Table 3 below summarizes the *mmms* in terms of their sequential position and key features.

**Table 3:** Summary of types of gustatory *mmm* during infant mealtimes.

**Type of gustatory *mmm***	**Typical sequential position**	**Key features**
Announcement	At start of meal or introduction of a food item	Parental eye gaze on the food or related objects Prior to infant feeding Standalone *mmm* or *mmm* + object-side assessment
Receipting	As infants' mouth closes around food item	Parental eye gaze on the infant At moment when food goes into mouth Using standalone *mmm*s
Modeling	At any point during the mealtime	Parental eye gaze on the infant Parents eating food Often exaggerated or extended *mmm* or combined with other lexical or non-lexical markers
Encouragement	At any point during the mealtime	Parental eye gaze on the infant Infant not actively chewing or eating Often exaggerated or extended *mmm* or combined with other lexical or non-lexical markers Often accompanied by verbal checks with regards to taste

Across all four types, some key findings can be summarized:

- Gustatory *mmms* during infant meals are predominantly standalone in first turn position
- The *mmm* + evaluation sequence was almost always with an object-side assessment
- Eye gaze was a central feature of the *mmm*s in that parental eye gaze was always focused on the child (or, in the case of the announcement *mmms*, on the food) at the start of the sound.

The regularity in the sequential positioning and organization within the social interaction are strong evidence that the *mmms* were not produced purely on the basis of, for example, olfactory, or gustatory senses of the parent (smelling or tasting of the food). Nor might the parents have been attending to the facial expressions of their infants, since the *mmm*s occurred in the corpus at the same sequential point relative to the food in the mouth, regardless of any facial expressions of the infants. They appear to be more closely tied to the sequentiality of the interaction than to individual characteristics. The potential “third position” of the receipting *mmms*, for instance, was particularly regular, in which the *mmm* occurred after the food was first carried to, then placed within, the mouth.

In contrast to the work discussed in the introduction, this paper argues that it is important to examine enjoyment as a socially normative practice *enacted within interaction*, and to observe when and how it occurs during mealtimes. It becomes relevant at certain moments—when food is being introduced, when food is placed in the mouth, when there is eye contact between parent and infant, and when there might be a need to encourage an infant to eat more food. The parents are not only attending to their own enjoyment (modeling mmm), they are also non-lexically embodying the assumed or potential sensory experiences that they might expect their infant to enjoy. Enjoyment can therefore be much more than an individual concept; it can be part of the glue that holds mealtimes together. As such, it need not be considered antagonistic to notions of health, since one might argue that the health of the infant is dependent in part on them consuming sufficient food. The gustatory *mmm* does not in itself specify whether or not something is “healthy” nor what it is that makes it pleasurable. As a non-lexical vocalization it is semantically flexible and thus provides for an orientation to enjoyment without precluding health. It does not, as it were, rely on the health vs. pleasure dichotomy.

The paper also provides a potential bridge between cultural and psychological work on eating enjoyment, focusing as it does on the interaction between parent and infant, and on those moments in which enjoyment becomes socially available. There are other connections, too. The announcement *mmms* are reminiscent of food advertising, for instance, in that they orient to the to-be-consumed food item immediately before it is offered to the infant. In a similar way, advertising tempts us through images of food before it is eaten; orienting to the anticipation of a meal before the appetite is sated (Korsmeyer and Sutton, 2011). They work rather differently, then, to those *mmms* which occur later in the meal, since they orient to enjoyment as encompassing the expectation of taste as much as they do of the taste itself. The use of modeling and encouragement *mmms* to engage infants in the eating process, whether or not the parents are themselves eating, also attends to the complex interplay between the social aspects of eating and the work of feeding infants.

There are limitations to this study that should be acknowledged. This was a study that used video-recordings taken by families in Scotland as examples of naturalistic meals in family homes. The families were not asked to feed in a specific manner, and therefore there is considerable variation across the corpus in terms of the feeding context (position of parent in relation to infant, use of utensils, and so on). The study was also limited in number of families: only five took part, and the ages of the infants varied from 5 to 8 months (even then, only the approximate age in months was recorded). The feeding of infants can change in important ways during these months and subtle variations might have been missed (cf. Negayama, 1993; van Dijk et al., 2012; Toyama, 2014). No other demographic information about the families other than the age of the parents was recorded. There was variation in the number of meals recorded, and in the timing of those meals throughout the day. In short, the data set represents a snapshot of a small cohort of families in Scotland, with limited demographic information upon which to catalog the sample. While this is counterbalanced by the repeated patterns found in the use of the non-lexical *mmm*, it is important to situate the findings within this research context.

While the research has met the study aims of examining how and where enjoyment becomes socially relevant in infant mealtimes, there is undoubtedly more work to be done. The gustatory *mmm* might be culturally normative within the English language, but research is needed into the use of similar non-lexical utterances in other languages and other mealtime contexts. The different types of *mmm*s classified in this paper would also benefit from further analysis: how they may be aligned with the progressivity of the meal, how might the modeling and encouragement *mmms* be further refined to distinguish between different activities as the meal progresses, and so on. The orientation to enjoyment as a cultural norm within other types of meals, with older children, or with only adults, would also be important to explore. What happens in those mealtimes which are more problematic or difficult for parents? We might then consider what happens when children are not eating at all, and what happens with the interaction during those occasions. As noted in the introduction, the cultural norm that meals should be enjoyable has not yet been matched by research to examine just how and when this enjoyment becomes an interactional practice, or what happens where this might be lacking.

## Data Availability

The datasets for this manuscript are not publicly available because the data was collected before open access protocols were in operation and the data comprises videos of infants in their homes. Requests to access the datasets should be directed to SW at sally.wiggins.young@liu.se.

## Ethics Statement

This study was carried out in accordance with the recommendations of the University of Strathclyde Psychology department ethics committee with written informed consent from all participants. All adult participants gave written informed consent in accordance with the Declaration of Helsinki. The protocol was approved by the University of Strathclyde Psychology department ethics committee.

## Author Contributions

The author was solely responsible for the design, data transcription and management, analysis, and writing of the paper.

### Conflict of Interest Statement

The author declares that the research was conducted in the absence of any commercial or financial relationships that could be construed as a potential conflict of interest.

## Footnotes

^1^Pseudonyms are provided for all infants, and parents are denoted by either ‘Mum’ or ‘Dad’ for ease of reference.

^2^Note that there were instances in the corpus where parents asked ‘what do you think?’ type questions at the point of feeding, which does orient to the possibility of infant input, whether verbally or non-verbally.
